# Depressive and anxiety disorders linked to bullying victimization in North Africa and the Middle East: An analysis based on sex and location

**DOI:** 10.1097/MD.0000000000045745

**Published:** 2025-11-14

**Authors:** Sohrab Amiri, Seyyed Mohammad Hossein Kazemi

**Affiliations:** a Spiritual Health Research Center, LifeStyle Institute, Baqiyatallah University of Medical Sciences, Tehran, Iran; b Student Research Committee, Baqiyatallah University of Medical Sciences, Tehran, Iran.

**Keywords:** anxiety, bullying victimization, depressive, Global Burden of Disease, mental disorder, North Africa and the Middle East

## Abstract

This research aimed to investigate the prevalence and disability caused by depressive disorder and anxiety disorders in North Africa and the Middle East (NAME). Depressive and anxiety disorders attributed to bullying victimization in NAME were also investigated. All age and Age-standardized disability-adjusted life years (DALYs) estimates of depressive and anxiety disorders attributed to bullying victimization were calculated, for the years 1990–2021. Estimates were based on per 100,000 populations. The 95% uncertainty interval was reported for each of the reported estimates. In 2021, the prevalence of depressive disorder was 31 million, and the prevalence of anxiety disorder was 38 million. The prevalence of depressive disorders and DALYs is higher in females than males. DALYs of depressive and anxiety disorders attributed to bullying victimization in NAME were 511,201 and 410,820 respectively in 2021. DALYs of depressive and anxiety disorders attributed to bullying victimization have a higher burden in males. In 2019, at the same time as the COVID-19 pandemic, there was a rapid growth in the prevalence of depressive disorders and anxiety disorders. There has been an increase in bullying victimization, and this has led to an increase in depressive and anxiety disorders attributed to bullying. Therefore, at the national level, it is necessary to make health policies on screening for mental disorders and educating children and adolescents in dealing with bullying victimization.

## 1. Introduction

Mental disorders are increasingly recognized as one of the main causes of disease burden.^[1]^ The Lancet Commission emphasizes mental health as a fundamental human right that is necessary for the development of all countries.^[1]^ Diagnosis and classification of mental disorders has been a challenging issue throughout history, and it was in the middle of the 20th century that systematic efforts were made to classify mental disorders.^[2]^ The most common way of classifying mental disorders is the International Classification of Diseases (ICD)^[3,4]^ developed by the World Health Organization, and the other is the diagnostic and statistical manual of mental disorders (DSM).^[5,6]^

In 2019, one out of 8 people, that is, 970 million people in the world, lived with a mental disorder, anxiety, and depressive disorders are the most common types of mental disorders among them.^[7]^ 301·4 million anxiety reported in 2019 globally, 113·9 million in males and 187·5 million in females.^[8]^ For depressive disorder, 279·6 million cases were reported, 109·2 million in males and 170·4 million in females.^[8]^ Age-standardized prevalence for anxiety disorders was 3779·5 per 100,000, and for depressive disorders was 3440·1.^[8]^ Risk factors for anxiety and depression are classified into biological, psychological, and social factors.^[9–14]^ One of the important and effective factors in mental disorders is adverse childhood experiences especially bullying victimization.^[15–18]^

In the Global Burden of Diseases (GBDs), one of the risk factors related to health issues is bullying victimization, conceptualized as “the intentional and repeated harm of a less powerful individual by peers.”^[19]^ Exposure to bullying victimization has many health consequences at different stages of life,^[20,21]^ including obesity,^[22]^ health risk behaviors,^[23]^ self-hram,^[24]^ and mental disorders.^[25]^ The study conducted at the global level has also examined mental disorders attributed to bullying victimization^[26]^ and the results obtained have shown that mental disorders attributed to bullying have increased compared to 1990.^[26]^

In some regions of the world, the prevalence of mental disorders has been reported to be higher, including North Africa and the Middle East (NAME).^[8]^ This region has been one of the conflict regions in recent decades, and this has also affected mental health. Conflict areas lead to an increase in migration, refugees, and asylum-seekers and this increases mental disorders.^[27,28]^ The study has shown that facing conflict is associated with an increase in anxiety disorders and depressive disorder.^[29]^ According to what was stated, this research aimed to investigate the prevalence and disability caused by depressive disorder and anxiety disorders in NAME. Depressive and anxiety disorders attributed to bullying victimization in NAME were also investigated. The estimates obtained in this research were based on the Global Burden of Diseases 2021 and included incidence, prevalence, and disability-adjusted life years (DALYs), stratified by sex and country. Also, the trends of depressive and anxiety disorders and exposure to bullying victimization between 1990 to 2021 were investigated.

## 2. Methods

### 2.1. Ethical review

This study is based on secondary analysis, with no new data collection involved, and therefore does not require ethical approval.

## 3. Involving human participants and/or animals

None applicable.

## 4. Informed consent

None applicable.

## 5. Data source

This research was based on the Global Burden of Disease 2021.^[19,30–33]^ GBD 2021 has been reporting incidence, prevalence, DALYs, years lived with disability (YLDs), years of life lost (YLLs), and death for 371 diseases and injuries along with estimates of healthy life expectancy (HALE). These estimates are provided for sex, and age groups and for 204 countries and territories, including sub-national estimates for 21 countries.^[30]^ Data sources used in GBD 2021 included 100,983 data sources (19,189 new data sources for DALYs) 12 new causes, and other important methodological updates.^[30]^ GBD 2021 risk factor analysis used data from 54,561 total distinct sources to produce epidemiological estimates for 88 risk factors and their associated health outcomes for a total of 631 risk–outcome pairs.^[19]^ More details about the data sources, and methodology of GBD 2021 are reported elsewhere.^[30]^

## 6. Case definitions

Depressive disorders in GBD 2021 include 2 subcategories Major depressive disorder (MDD) and Dysthymia.^[30]^ Major depressive disorder (MDD) is defined as “an episodic mood disorder involving the experience of one or more major depressive episode(s). Included in the GBD disease modeling were cases meeting diagnostic criteria for MDD according to the DSM or the equivalent diagnosis of recurrent depression in the ICD.^[4,34]^ These were identified by the following codes: DSM-IV-TR: 296.21–24, 296.31–34; ICD-10: F32.0–9, F33.0–9; excluding those cases due to a general medical condition or substance-induced cases.^[4,34]^ Different versions of DSM (DSM-III, DSM-III-R, DSM-IV, DSM-IV-TR, DSM-5, and DSM-5-TR) and ICD (ICD-9, ICD-10, and ICD-11) were accepted.”^[30]^ Dysthymia is defined as “a mood disorder consisting of chronic depression, demonstrating less severe but longer lasting symptoms than the major depressive disorder. Included in GBD disease modeling were cases meeting diagnostic criteria for dysthymia according to the DSM, or the equivalent diagnosis in the ICD.^[4,34]^ These were identified by the following codes: DSM-IV-TR: 300.4, ICD-10: F34.1; excluding those cases due to a general medical condition or substance-induced cases.^[4,34]^ Different versions of DSM (DSM-III, DSM-III-R, DSM-IV, DSM-IV-TR, DSM-5, and DSM-5-TR) and ICD (ICD-9, ICD-10, and ICD-11) were accepted.”^[30]^

Anxiety disorders are defined as “experiences of intense fear and distress, typically in combination with other physiological symptoms. These diagnoses were based on the DSM or the equivalent diagnosis of recurrent depression in the ICD.^[4,34]^ Anxiety disorders included panic disorder, agoraphobia, specific phobia, social phobia, obsessive-compulsive disorder, post-traumatic stress disorder, generalized anxiety disorder including overanxious disorder in childhood, separation anxiety disorder, and anxiety disorder ‘not otherwise specified’ (NOS). These were identified by the following codes: DSM-IV-TR: 300.0–300.3, 208.3, 309.21, 309.81; ICD-10: F40–42, F43.0, F43.1, F93.0–93.2, F93.8. Excluded were anxiety disorders due to a general medical condition and substance-induced anxiety disorder. Different versions of DSM (DSM-III, DSM-III-R, DSM-IV, DSM-IV-TR, DSM-5, and DSM-5-TR) and ICD (ICD-9, ICD-10, and ICD-11) were accepted.”^[30]^

Bullying victimization is defined as “the intentional and repeated harm of a less powerful individual by peers.”^[35]^ The case definition in GBD for bullying victimization is “bullying victimization of children and adolescents attending school by peers. This definition includes the global concept of bullying victimization, which incorporates combined estimates of subtypes such as physical, verbal, relational, and cyberbullying victimization. It excludes abuse/harassment by siblings, intimate partners, and adults (e.g., teachers). While bullying can be experienced as either a victim or perpetrator, perpetration (i.e., those who bully others) is not included in this definition although some victims will also be perpetrators”.^[19]^ Refer elsewhere for more details on GBD methodology.^[19,30–33]^

## 7. Estimation framework

Years lived with disability (YLDs) were estimated by multiplying prevalence estimates at varying levels of severity by an appropriate disability weight.^[30]^ YLLs were calculated by multiplying cause-specific deaths by the years of life expected to remain at death based on a normative life expectancy.^[30]^ DALYs were calculated as the sum of YLDs and YLLs.^[30]^

## 8. Statistics

In GBD 2021, the relationship between 88 risk factors with selected health outcomes has been estimated.^[19]^ All age and age-standardized incidence, prevalence, and DALYs, for depressive and anxiety disorders were calculated and results were also stratified by country and sex. For estimation of the association between bullying victimization and depressive and anxiety disorders, all age and age-standardized DALY estimates of depressive and anxiety disorders attributed to bullying victimization were calculated, for the years 1990–2021^[19]^; results were also stratified by country and sex. Estimates were based on age-standardized rate estimates (per 100,000), all-ages rate estimates (per 100,000), and all-ages count estimates. The 95% uncertainty interval was reported for each of the reported estimates. More details about data, data processing, and modeling are elsewhere which are related to GBD 2021.^[19,30]^ Summary exposure value (SEV) is the RR-weighted prevalence of exposure, a univariate measure of risk-weighted exposure, taking the value zero when no excess risk for a population exists and the value 1 when the population is at the highest level of risk. We report SEVs on a scale from 0 to 100% on which a decline in SEV indicates reduced exposure to a given risk factor and an increase in SEV indicates increased exposure.^[19]^

GBD 2021 complies with the Guidelines for Accurate and Transparent Health Estimates Reporting^[36]^; Analyses were completed using Python (version 3.10.4), Stata (version 13.1), and R (version 4.2.1).

### 8.1. Results

#### 8.1.1. Prevalence, incidence, and DALYs of depressive disorder in North Africa and the Middle East

**Figure 1.**
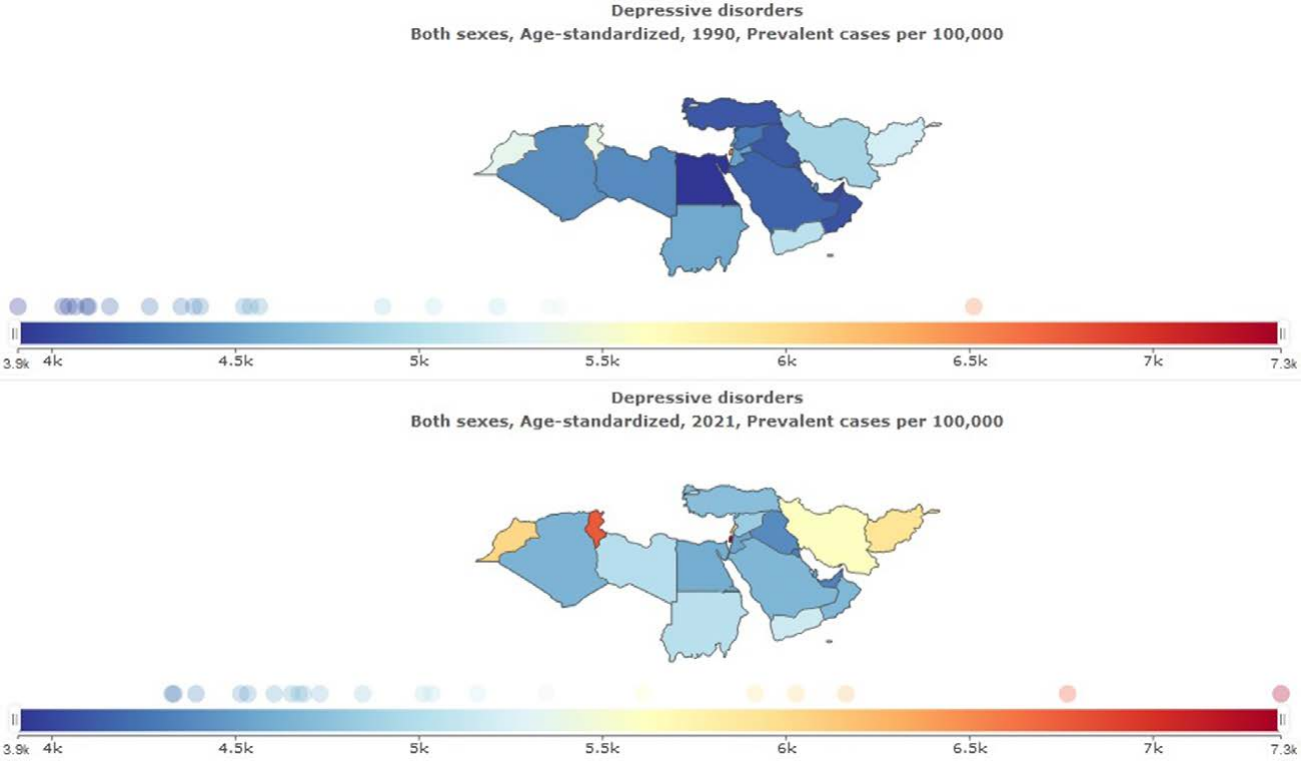
Age-standardized prevalence ratio of depressive disorders in North Africa and the Middle East, 1990–2021.

**Figure 2.**
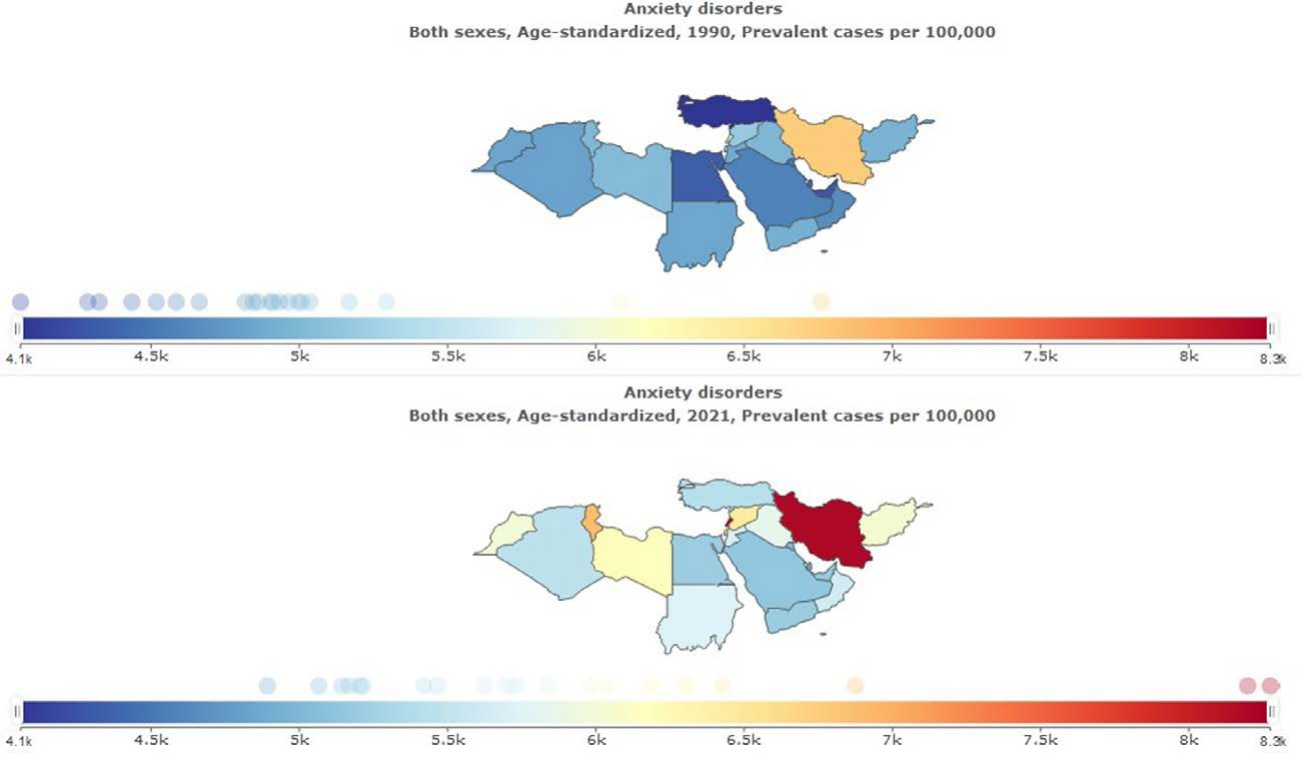
Age-standardized prevalence ratio of anxiety disorders in North Africa and the Middle East, 1990–2021.

**Figure 3.**
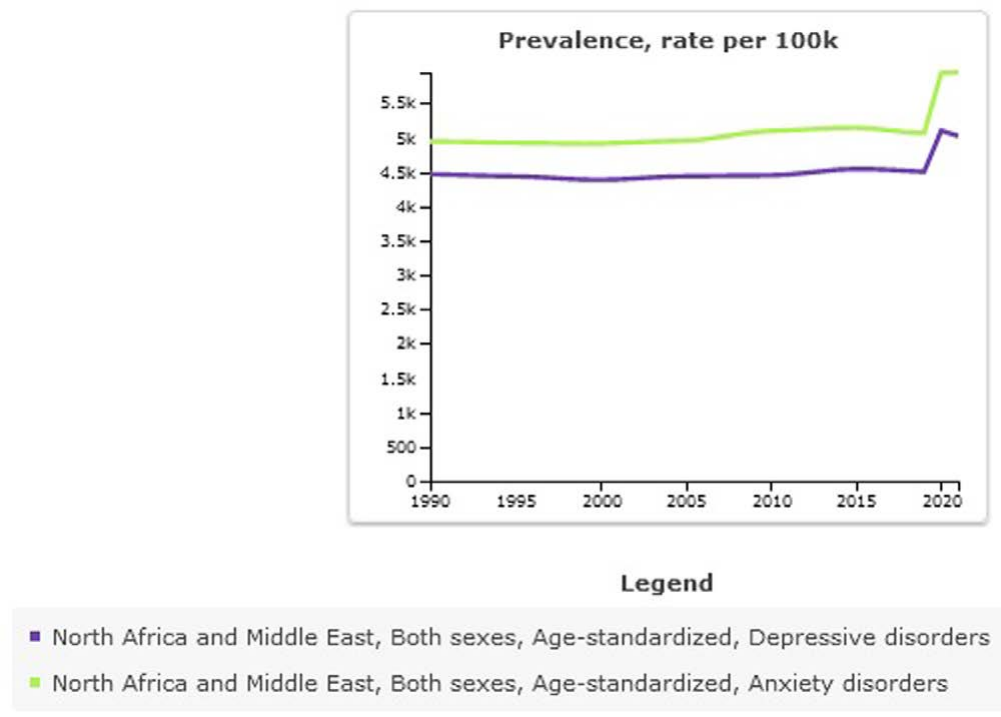
Trend of age-standardized prevalence ratio of depressive and anxiety disorders in North Africa and the Middle East stratified, 1990–2021.

In 2021, the prevalence of depressive disorder was 31,377,638 (95% UI: 26,947,181–36,911,406) in NAME (Table 1). This represents a significant increase compared to 1990 when depressive disorder was 12,447,648 (95% UI: 10,794,844–14,728,647). Age-standardized prevalence rate per 100,000 for depressive disorder was 5024 (95% UI: 4346–5857) in 2021, percentage change from 1990 to 2021 was 0.12 (95% UI: 0.07–0.18) (Figs. 1–3).

**Table 1:** Prevalence, Incidence, DALYs, of depressive and anxiety disorders in North Africa and the Middle East, 1990–2021.

Measure	Cause	Year								
		1990			2021			Percentage change 1990–2021		
		Value	Lower	Upper	Value	Lower	Upper	Value	Lower	Upper
Age-standardized rate estimates (per 100,000)										
Prevalence	Depressive disorders	4468.97	3904.16	5216.63	5024.69	4346.39	5857.38	0.12	0.07	0.18
Incidence	Depressive disorders	5138.95	4371.20	6131.27	5983.10	4953.30	7214.68	0.16	0.1	0.24
DALYs	Depressive disorders	788.13	536.47	1078.26	900.68	598.46	1242.73	0.14	0.08	0.21
Prevalence	Anxiety disorders	4945.43	4195.92	5812.07	5950.26	4892.21	7231.97	0.2	0.12	0.28
Incidence	Anxiety disorders	728.07	606.87	902.13	883.41	722.42	1108.54	0.21	0.12	0.3
DALYs	Anxiety disorders	587.75	407.24	806.3	707.08	469.77	994.9	0.2	0.12	0.28
All-ages rate estimates (per 100,000)										
DALYs	Depressive disorders	657.75	444.43	909.29	908.27	599.25	1248.14	0.38	0.3	0.46
Prevalence	Depressive disorders	3669.80	3182.52	4342.28	5036.54	4325.39	5924.78	0.37	0.3	0.45
Incidence	Depressive disorders	4326.04	3644.75	5236.20	6018.78	4942.31	7334.25	0.39	0.3	0.49
DALYs	Anxiety disorders	562.79	387.13	791.31	723.61	476.6	1022.20	0.29	0.19	0.39
Prevalence	Anxiety disorders	4682.11	3913.99	5627.08	6067.37	4962.70	7430.08	0.3	0.2	0.4
Incidence	Anxiety disorders	761.57	621.13	935.63	915.38	745.43	1150.47	0.2	0.11	0.3
All-ages counts estimates										
DALYs	Depressive disorders	2,231,029.55	1,507,486.06	3,084,241.83	5,658,552.48	3,733,340.89	7,775,915.42	1.54	1.38	1.69
Prevalence	Depressive disorders	12,447,648.53	10,794,844.93	14,728,647.98	31,377,638.15	26,947,181.21	36,911,406.83	1.52	1.38	1.66
Incidence	Depressive disorders	14,673,569.47	12,362,678.81	17,760,762.13	37,496,992.97	30,790,569.31	45,692,398.06	1.56	1.38	1.73
DALYs	Anxiety disorders	1,908,924.92	1,313,103.31	2,684,061.16	4,508,067.43	2,969,187.28	6,368,320.64	1.36	1.18	1.55
Prevalence	Anxiety disorders	15,881,329.48	13,275,923.77	19,086,607.03	37,799,731.40	30,917,632.50	46,289,420.78	1.38	1.21	1.57
Incidence	Anxiety disorders	2,583,166.97	2,106,820.55	3,173,584.03	5,702,847.41	4,644,051.75	7,167,442.13	1.21	1.03	1.4

#### 8.1.2. Prevalence, incidence, and DALYs of anxiety disorder in North Africa and the Middle East

In 2021, the prevalence of anxiety disorder was 37,799,731 (95% UI: 30,917,632–46,289,420) in NAME (Table 1). This represents a significant increase compared to 1990 when anxiety disorder was 15,881,329 (95% UI: 13,275,923–19,086,607). Age-standardized prevalence rate per 100,000 for anxiety disorder was 5950 (95% UI: 4892–7231) in 2021, percentage change 1990 to 2021 was 0.20 (95% UI: 0.12–0.28) (Figs. 2 and 3).

#### 8.1.3. The burden of depressive and anxiety disorder in North Africa and the Middle East is stratified by male and female

**Figure 4.**
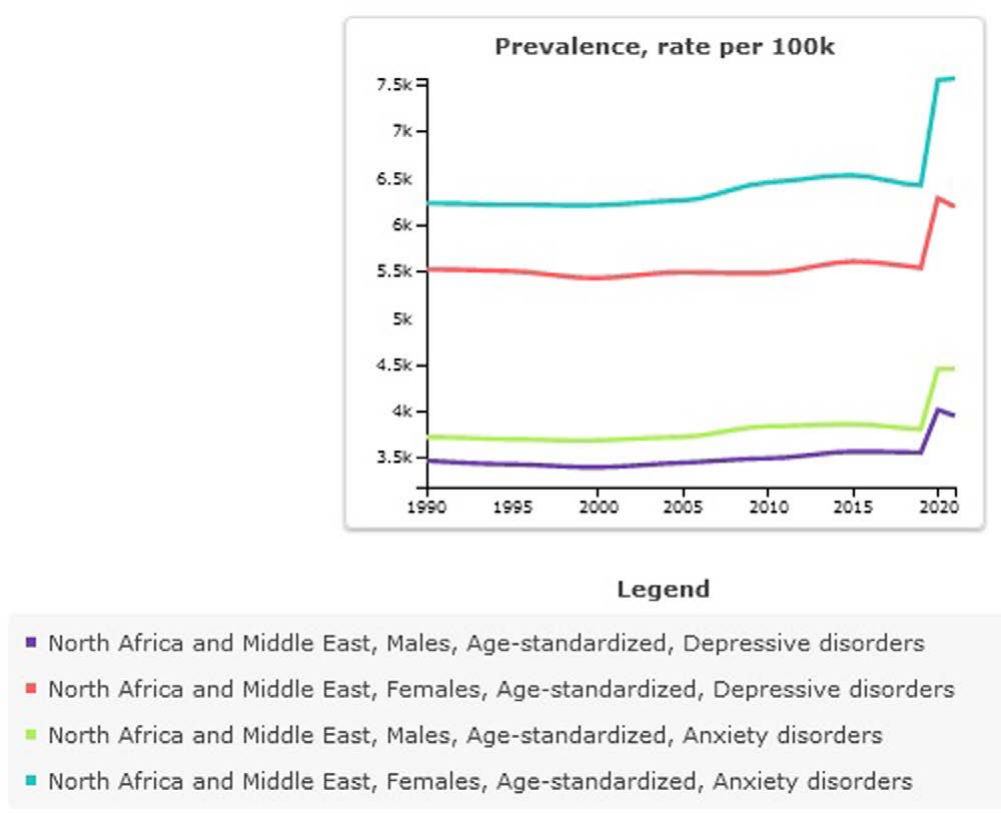
Age-standardized prevalence ratio of depressive and anxiety disorders in North Africa and the Middle East stratified by Sex, 1990–2021.

In 2021, the age-standardized prevalence rate per 100,000 for depressive disorders in males was 3949 (95% UI: 3445–4569), and in females was 6190 (95% UI: 5312–7265). In both sexes, there is an increase in depressive disorder and anxiety disorders compared to 1990 (95% UI: 0.14 vs 0.12). In 2021, the age-standardized prevalence rate per 100,000 for anxiety disorders in males was 4452 (95% UI: 3641–5440), and in females was 7566 (95% UI: 6174–9273). In both sexes, there is an increase in depressive disorder and anxiety disorders compared to 1990 (0.20 vs 0.21). The prevalence and disability caused by depressive disorder and anxiety disorders are higher in women compared to men (Table S1, Supplemental Digital Content, https://links.lww.com/MD/Q576, Fig. 4).

#### 8.1.4. Exposure to bullying victimization in North Africa and the Middle East

**Figure 5.**
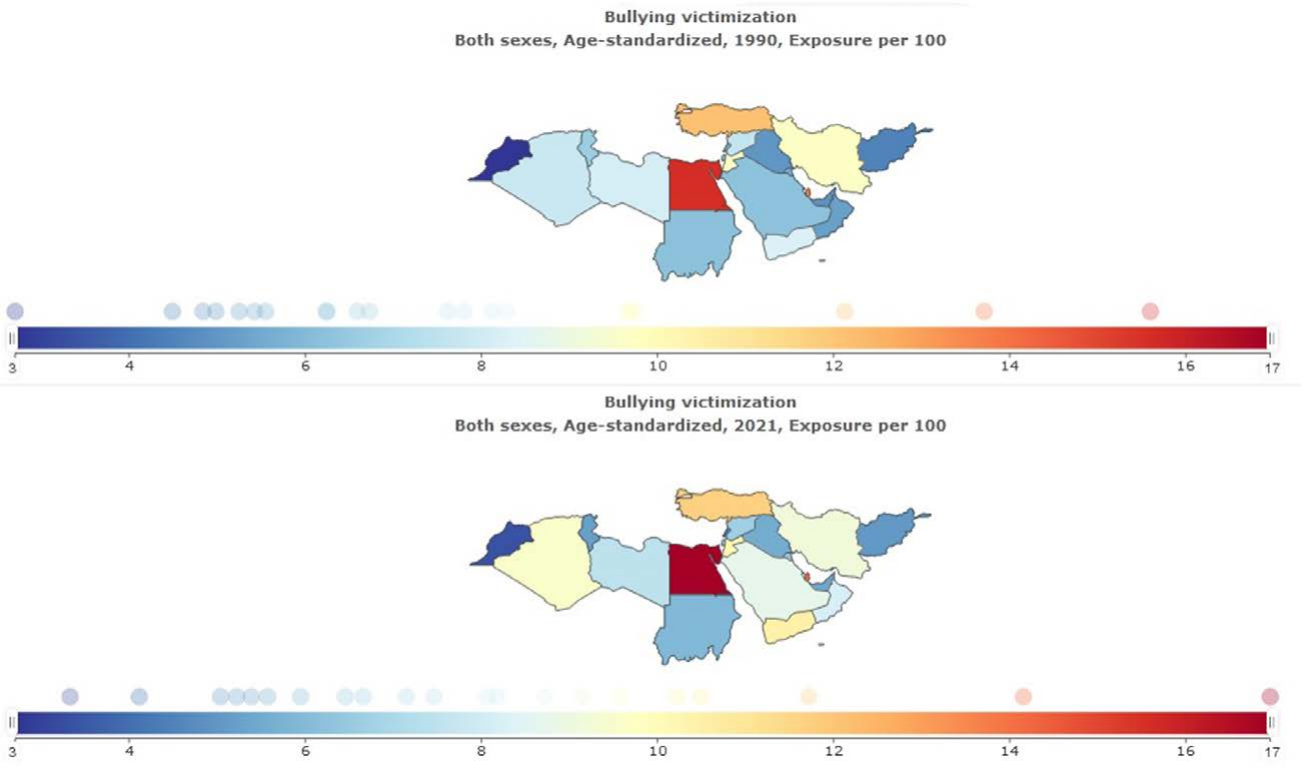
Summary exposure value for bulling victimization in North Africa and the Middle East stratified by country, 1990–2021.

**Figure 6.**
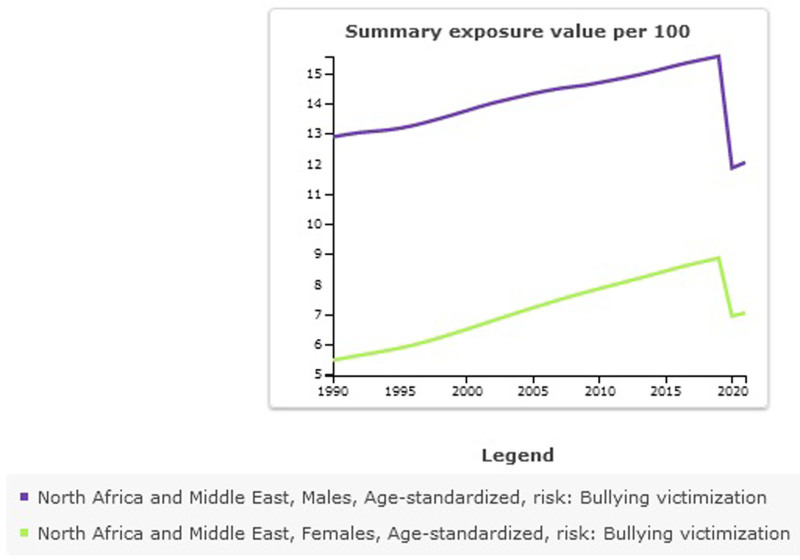
Trend of Summary exposure value for bulling victimization in North Africa and the Middle East stratified by sex, 1990–2021.

SEV per 100 for bullying victimization in NAME in 2021 was 9.64 (95% UI: 4.57–16.6). The highest age-standardized SEV was for Egypt, 16.96 (95% UI: 8.6–28.42), and the lowest was for Morocco, 3.33 (95% UI: 1.69–6.26). The highest percentage change from 1990 to 2021 was in Oman 0.56 (95% UI: 0.11–1.28) (Table 2, Fig. 5). Exposure to bullying victimization decreased in males −0.07 (95% UI: −0.21–0.17) and increased in females 0.29 (95% UI: 0.06–0.64) between 1990 and 2021 (Table 3, Fig. 6).

**Table 2:** Summary exposure value for bulling victimization in North Africa and the Middle East stratified by country, 1990–2021.

Location	Year								
	1990			2021			Percentage change 1990–2021		
	Value	Lower	Upper	Value	Lower	Upper	Value	Lower	Upper
Age-standardized									
North Africa and Middle East	9.27	4.14	17.87	9.64	4.57	16.6	0.04	−0.12	0.3
Afghanistan	4.49	1.94	8.81	5.04	2.08	10.02	0.12	−0.13	0.47
Algeria	7.81	3.43	15.38	9.57	4.48	18.08	0.23	−0.09	0.65
Bahrain	9.68	4.1	18.99	8.06	3.44	16.33	−0.17	−0.31	0.03
Egypt	15.6	7.03	28.03	16.96	8.6	28.42	0.09	−0.25	0.62
Iran (Islamic Republic of)	9.67	4.22	18.74	9.15	4.29	16.62	−0.05	−0.15	0.09
Iraq	4.99	2.17	9.52	5.57	2.61	10.81	0.12	−0.11	0.51
Jordan	9.74	4.19	18.4	10.22	4.98	18.91	0.05	−0.25	0.48
Kuwait	5.55	2.3	11.11	6.45	2.94	12.28	0.16	−0.15	0.61
Lebanon	5.42	2.3	10.46	4.11	2.08	7.4	−0.24	−0.47	0.12
Libya	8.12	3.57	16.19	7.46	3.59	14.57	−0.08	−0.29	0.23
Morocco	2.7	1.14	5.41	3.33	1.69	6.26	0.23	−0.15	0.77
Oman	5.25	2.27	10.6	8.18	3.93	15	0.56	0.11	1.28
Palestine	6.73	2.95	13.24	7.15	2.95	13.99	0.06	−0.17	0.35
Qatar	13.71	5.92	27.18	14.16	6.68	26.39	0.03	−0.22	0.44
Saudi Arabia	6.25	2.75	12.15	8.73	3.56	17.43	0.4	0.1	0.74
Sudan	6.24	2.61	11.87	5.95	2.54	11.86	−0.05	−0.26	0.19
Syrian Arab Republic	7.62	3.31	14.51	6.66	2.89	13.1	−0.13	−0.3	0.08
Tunisia	6.59	2.82	12.72	5.22	2.53	10.01	−0.21	−0.38	0.06
Türkiye	12.13	5.4	23.65	11.72	5.82	20.7	−0.03	−0.31	0.39
United Arab Emirates	4.84	2.09	9.6	5.39	2.71	9.77	0.11	−0.2	0.61
Yemen	8.28	3.53	16.3	10.5	4.76	19.92	0.27	−0.03	0.68
All ages									
North Africa and Middle East	11.11	5.06	21.24	10.19	4.8	17.55	−0.08	−0.23	0.16
Afghanistan	5.25	2.37	9.99	6.29	2.76	12.43	0.2	−0.08	0.59
Algeria	9.67	4.25	18.73	9.39	4.37	17.65	−0.03	−0.27	0.29
Bahrain	11.62	4.82	23.06	8.5	3.45	17.61	−0.27	−0.4	−0.1
Egypt	18.52	8.5	33.01	18.68	9.56	31.04	0.01	−0.3	0.51
Iran (Islamic Republic of)	11.42	5.1	21.84	8.74	4.06	16.14	−0.24	−0.32	−0.13
Iraq	6.11	2.76	11.71	6.5	3.07	12.55	0.06	−0.16	0.41
Jordan	12.72	5.67	24.29	11.89	5.81	21.95	−0.07	−0.32	0.32
Kuwait	6.65	2.72	13.34	6.23	2.63	12.21	−0.06	−0.31	0.26
Lebanon	5.99	2.58	11.58	4.09	2.02	7.49	−0.32	−0.52	0
Libya	10.16	4.59	20.09	7.9	3.74	15.61	−0.22	−0.41	0.04
Morocco	3.26	1.41	6.52	3.36	1.69	6.34	0.03	−0.29	0.49
Oman	6.13	2.68	12.17	8.82	4.08	16.57	0.44	−0.02	1.11
Palestine	8.4	3.82	16.32	8.64	3.63	16.78	0.03	−0.2	0.31
Qatar	15.84	6.57	31.77	15.2	6.7	29.56	−0.04	−0.27	0.32
Saudi Arabia	7.62	3.41	14.77	9.27	3.61	18.99	0.22	−0.06	0.55
Sudan	7.5	3.19	14.26	7.37	3.23	14.65	−0.02	−0.24	0.22
Syrian Arab Republic	9.48	4.28	17.84	7.26	3.19	14.21	−0.23	−0.39	−0.04
Tunisia	7.87	3.4	15.11	4.76	2.24	9.25	−0.4	−0.53	−0.19
Türkiye	14.48	6.49	28.1	11.09	5.42	19.65	−0.23	−0.45	0.1
United Arab Emirates	5.47	2.32	10.83	4.47	2.05	8.79	−0.18	−0.44	0.17
Yemen	9.58	4.28	18.37	12.91	5.95	24.41	0.35	0.03	0.78

**Table 3:** Summary exposure value for bulling victimization in North Africa and the Middle East stratified by sex, 1990–2021.

Metric	Sex	Location	1990			2021			Percentage change 1990–2021		
			Value	Lower	Upper	Value	Lower	Upper	Value	Lower	Upper
Age-standardized	Male	North Africa and Middle East	12.89	5.71	24.33	12.04	5.8	20.22	−0.07	−0.21	0.17
		Afghanistan	7.53	3.26	14.93	7.34	3.05	14.53	−0.02	−0.24	0.27
		Algeria	9.43	4.02	18.74	9.98	4.74	19	0.06	−0.21	0.47
		Bahrain	12.18	5.14	24.16	9.72	4.25	19.57	−0.2	−0.35	0
		Egypt	20.39	9.25	35.73	19.2	9.88	31.27	−0.06	−0.37	0.44
		Iran (Islamic Republic of)	15.26	6.58	29.38	13.88	6.47	24.03	−0.09	−0.21	0.08
		Iraq	7.36	3.16	14.19	7.44	3.49	14.31	0.01	−0.21	0.36
		Jordan	12.96	5.61	25.04	12.91	6.48	23.45	0	−0.3	0.47
		Kuwait	8.39	3.48	16.97	9.82	4.42	18.94	0.17	−0.14	0.63
		Lebanon	8.33	3.55	16.51	5.73	2.93	10.52	−0.31	−0.52	0.05
		Libya	11.27	4.96	22.44	9.86	4.75	19.25	−0.13	−0.34	0.19
		Morocco	3.16	1.34	6.34	3.36	1.73	6.19	0.06	−0.3	0.62
		Oman	6.4	2.79	12.94	8.97	4.49	16.24	0.4	−0.02	1.1
		Palestine	9.1	3.95	17.66	9.14	3.8	17.91	0	−0.23	0.32
		Qatar	15.28	6.68	30.29	15.29	7.27	28.37	0	−0.26	0.41
		Saudi Arabia	8.4	3.64	16.11	10.91	4.45	21.85	0.3	0	0.64
		Sudan	9.32	4.01	17.28	8.07	3.48	15.96	−0.13	−0.34	0.11
		Syrian Arab Republic	10.91	4.69	21.24	9.15	4.05	17.69	−0.16	−0.34	0.06
		Tunisia	9.9	4.22	19.01	7.21	3.58	13.75	−0.27	−0.44	−0.03
		Türkiye	16.34	7.23	31.63	14.39	7.38	24.95	−0.12	−0.39	0.3
		United Arab Emirates	5.96	2.62	11.95	6.55	3.41	11.79	0.1	−0.25	0.63
		Yemen	12.9	5.52	24.66	14.95	6.83	28.24	0.16	−0.12	0.55
	Female	North Africa and Middle East	5.47	2.4	10.59	7.04	3.29	12.49	0.29	0.06	0.64
		Afghanistan	1.97	0.75	3.95	2.6	1.06	5.36	0.32	−0.13	0.96
		Algeria	6.14	2.65	11.97	9.17	4.06	17.45	0.49	0.1	1
		Bahrain	6.23	2.61	12.27	5.33	2.11	10.92	−0.15	−0.28	0.02
		Egypt	10.54	4.6	20.74	14.58	6.98	24.9	0.38	−0.08	1.07
		Iran (Islamic Republic of)	3.95	1.77	7.68	4.21	1.9	8.47	0.07	−0.05	0.22
		Iraq	2.42	1.06	4.7	3.54	1.59	6.71	0.46	0.12	0.96
		Jordan	6.13	2.5	12.3	7.07	3.15	13.39	0.15	−0.15	0.62
		Kuwait	2.07	0.88	4.06	2.91	1.37	5.37	0.41	0.01	0.98
		Lebanon	2.62	1.11	5.18	2.37	1.13	4.29	−0.1	−0.37	0.31
		Libya	4.59	1.96	8.93	4.93	2.28	9.5	0.07	−0.15	0.38
		Morocco	2.25	0.92	4.56	3.31	1.62	6.48	0.47	0	1.16
		Oman	3.39	1.5	6.64	6.8	3.11	12.83	1.01	0.37	1.93
		Palestine	4.3	1.79	8.69	5.08	2.02	9.98	0.18	−0.07	0.52
		Qatar	10.5	4.34	20.95	11.58	5.4	21.9	0.1	−0.17	0.54
		Saudi Arabia	3.42	1.4	6.97	5.79	2.21	11.61	0.69	0.34	1.15
		Sudan	3.25	1.29	6.52	3.82	1.62	7.67	0.18	−0.12	0.61
		Syrian Arab Republic	4.2	1.79	8.19	4.67	1.94	9.51	0.11	−0.11	0.37
		Tunisia	3.23	1.35	6.41	3.23	1.44	6.47	0	−0.22	0.35
		Türkiye	7.74	3.34	15.3	8.95	4.18	16.82	0.16	−0.16	0.61
		United Arab Emirates	2.95	1.26	5.83	3.58	1.68	6.67	0.21	−0.09	0.65
		Yemen	3.59	1.43	7.59	5.99	2.62	11.79	0.67	0.19	1.43
All ages	Male	North Africa and Middle East	15.22	6.94	28.74	12.75	6.13	21.5	−0.16	−0.29	0.04
		Afghanistan	8.26	3.79	16.05	9.09	4.02	17.64	0.1	−0.16	0.46
		Algeria	11.48	5.01	22.58	9.9	4.67	18.71	−0.14	−0.36	0.16
		Bahrain	14.43	6.06	28.89	10.19	4.15	20.75	−0.29	−0.42	−0.12
		Egypt	24.02	11.09	41.37	20.93	10.91	33.88	−0.13	−0.41	0.33
		Iran (Islamic Republic of)	17.71	7.81	34.22	13.48	6.23	23.72	−0.24	−0.34	−0.12
		Iraq	8.92	3.94	17.03	8.64	4.1	16.52	−0.03	−0.24	0.3
		Jordan	16.76	7.49	32.03	14.96	7.5	27.2	−0.11	−0.38	0.29
		Kuwait	9.64	3.92	19.6	9.39	3.96	18.6	−0.03	−0.27	0.32
		Lebanon	9.14	3.98	17.87	5.99	3	11.06	−0.34	−0.55	−0.02
		Libya	13.82	6.24	27.47	10.5	4.92	20.66	−0.24	−0.43	0.04
		Morocco	3.76	1.62	7.41	3.42	1.75	6.34	−0.09	−0.4	0.38
		Oman	7.38	3.24	14.88	9.78	4.57	18.58	0.33	−0.11	0.99
		Palestine	11.33	5.13	21.82	10.96	4.65	21.26	−0.03	−0.27	0.26
		Qatar	17.53	7.28	35.78	16.58	7.19	32.22	−0.05	−0.29	0.31
		Saudi Arabia	10.09	4.43	19.32	11.52	4.42	23.7	0.14	−0.13	0.47
		Sudan	10.87	4.79	20.02	9.82	4.36	19.31	−0.1	−0.32	0.16
		Syrian Arab Republic	13.25	5.89	25.62	9.48	4.22	18	−0.28	−0.44	−0.09
		Tunisia	11.66	5.08	22.37	6.7	3.24	12.92	−0.42	−0.56	−0.24
		Türkiye	19.39	8.68	37.36	13.97	7.07	24.51	−0.28	−0.5	0.07
		United Arab Emirates	6.49	2.74	13.05	4.99	2.24	9.92	−0.23	−0.49	0.14
		Yemen	14.69	6.71	27.78	18.14	8.35	34.31	0.23	−0.07	0.63
	Female	North Africa and Middle East	6.79	3.04	13.04	7.42	3.47	13.17	0.09	−0.1	0.41
		Afghanistan	2.41	0.92	4.79	3.38	1.41	6.89	0.4	−0.11	1.1
		Algeria	7.82	3.48	15.07	8.87	3.9	16.85	0.13	−0.16	0.52
		Bahrain	7.7	3.22	15.2	5.6	2.17	11.63	−0.27	−0.39	−0.13
		Egypt	12.74	5.63	24.58	16.27	7.86	27.6	0.28	−0.15	0.91
		Iran (Islamic Republic of)	4.89	2.26	9.49	3.84	1.69	7.86	−0.22	−0.32	−0.1
		Iraq	3.12	1.4	6.04	4.2	1.91	7.95	0.35	0.03	0.8
		Jordan	8.29	3.5	16.6	8.35	3.73	15.8	0.01	−0.25	0.4
		Kuwait	2.61	1.1	5.13	2.68	1.18	5.14	0.03	−0.26	0.42
		Lebanon	2.91	1.24	5.74	2.19	1.03	4.05	−0.25	−0.48	0.09
		Libya	6.15	2.72	11.81	5.14	2.33	10.03	−0.16	−0.35	0.09
		Morocco	2.77	1.14	5.59	3.3	1.6	6.48	0.19	−0.18	0.74
		Oman	4.3	1.96	8.13	7.26	3.29	13.84	0.69	0.13	1.47
		Palestine	5.47	2.35	10.85	6.23	2.51	12.2	0.14	−0.1	0.46
		Qatar	12.31	5.1	24.65	11.8	5.19	22.93	−0.04	−0.26	0.29
		Saudi Arabia	4.43	1.87	8.88	6.07	2.22	12.6	0.37	0.04	0.75
		Sudan	4.09	1.64	8.18	4.83	2.07	9.64	0.18	−0.13	0.63
		Syrian Arab Republic	5.55	2.43	10.67	5.22	2.21	10.57	−0.06	−0.24	0.17
		Tunisia	3.98	1.68	7.89	2.84	1.23	5.65	−0.29	−0.46	−0.04
		Türkiye	9.44	4.13	18.81	8.19	3.77	15.5	−0.13	−0.37	0.21
		United Arab Emirates	3.55	1.53	7.03	3.09	1.41	5.81	−0.13	−0.37	0.21
		Yemen	4.27	1.77	8.81	7.59	3.34	15	0.77	0.28	1.58

#### 8.1.5. Depressive disorders attributed to bullying victimization

**Figure 7.**
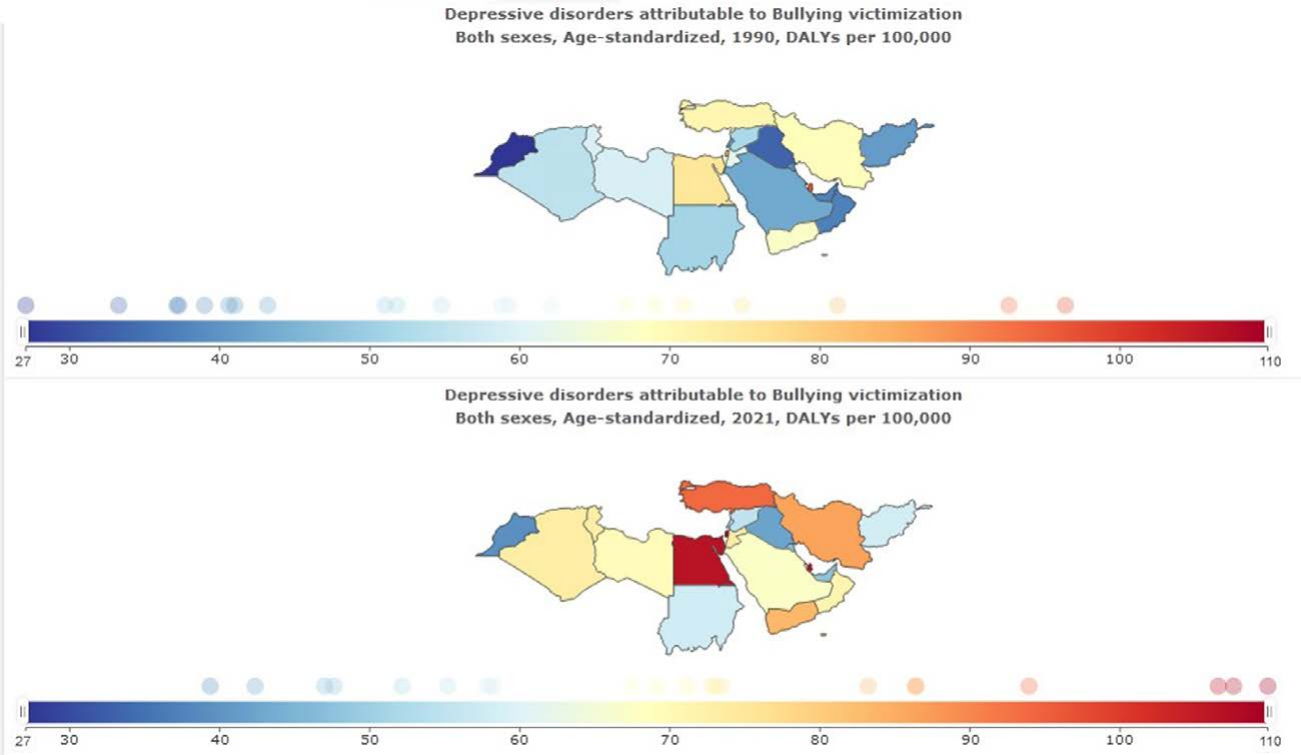
Depression disorders attributed to bulling victimization in North Africa and the Middle East stratified by country, 1990–2021.

DALYs of depressive disorders attributed to bullying victimization in NAME was 511,201 (95% UI: 224,528–926,816) in 2021; compared to 1990, it showed an increase of more than 2 times 215,015 (95% UI: 89,809–400,651). Age-standardized DALYs of depressive disorders rate attributed to bullying victimization per 100,000 was 76 (95% UI: 33–137) in 2021. The highest DALYs of depressive disorders attributed to bullying victimization in NAME was in Palestine, 109 (95% UI: 42–216) per 100,000. The lowest DALYs of depressive disorders attributed to bullying victimization in NAME was in Morocco, 39 (95% UI: 15–76) per 100,000 (Table 4, Fig. 7).

**Table 4:** DALYs of depressive disorders attributed to bulling victimization in North Africa and the Middle East stratified by country, 1990–2021.

Location	Year								
	1990			2021			Percentage change 1990–2021		
	Value	Lower	Upper	Value	Lower	Upper	Value	Lower	Upper
Age-standardized rate estimates (per 100,000)									
North Africa and Middle East	59.18	24.08	111.57	76.72	33.95	137.95	0.3	0.17	0.52
Afghanistan	41	15.14	83.93	58.21	21.47	119.92	0.42	0.21	0.69
Algeria	54.76	20.71	110.03	73	30.46	136.5	0.33	0.13	0.69
Bahrain	92.6	36.4	180.42	86.41	33.43	167.72	−0.07	−0.15	0.04
Egypt	74.8	30.82	132.43	106.55	49.87	180.05	0.42	0.2	0.82
Iran (Islamic Republic of)	69.01	27.9	130.12	86.35	36.18	156.46	0.25	0.17	0.38
Iraq	33.27	12.17	69.06	42.35	17.38	84.2	0.27	0.1	0.59
Jordan	62.1	23.53	121.52	73.44	32.49	136.25	0.18	−0.05	0.58
Kuwait	38.99	14.02	77.67	46.98	18.45	91.28	0.21	0.02	0.53
Lebanon	40.62	14.98	82.76	52.16	21.44	99.36	0.28	−0.06	0.74
Libya	58.8	22.49	118.77	69.17	28.91	134.81	0.18	0.02	0.43
Morocco	27.07	9.09	58.61	39.36	15.7	76.15	0.45	0.09	0.99
Oman	37.13	13.75	75.87	71.14	30.29	131.7	0.92	0.49	1.72
Palestine	81.18	30.96	157.52	109.84	42.7	216.8	0.35	0.17	0.55
Qatar	96.35	38.9	183.85	107.56	44.48	196.9	0.12	−0.03	0.33
Saudi Arabia	43.19	16.51	89.59	67.44	26.19	129.42	0.56	0.38	0.82
Sudan	51.02	19.87	97.64	57.84	21.84	115.39	0.13	−0.01	0.3
Syrian Arab Republic	51.81	20.11	100.56	55.19	21.2	108.15	0.07	−0.03	0.16
Tunisia	59.26	22.38	119.63	72.74	29.84	140.41	0.23	0.06	0.49
Türkiye	70.93	30.72	133.91	93.93	46.08	166.98	0.32	0.07	0.78
United Arab Emirates	37.26	13.51	78.48	47.6	20.44	89.74	0.28	−0.05	0.81
Yemen	67.16	25.23	127.5	83.21	35.32	154.17	0.24	0.07	0.54
All-ages rate estimates (per 100,000)									
North Africa and Middle East	63.39	26.48	118.12	82.05	36.04	148.77	0.29	0.17	0.53
Afghanistan	38.25	14.14	76.71	62.42	24.44	123.86	0.63	0.36	0.96
Algeria	60.14	23.16	118.25	72.85	29.97	137.17	0.21	0	0.54
Bahrain	115.88	46.04	227.55	103	39.18	203.51	−0.11	−0.21	0.02
Egypt	80.75	34.6	145.38	113.33	53.28	188.23	0.4	0.18	0.81
Iran (Islamic Republic of)	70.34	28.66	131.28	90.15	37.45	164.97	0.28	0.11	0.49
Iraq	35.39	13.45	72.29	48.04	20.05	94.63	0.36	0.16	0.7
Jordan	71.13	27.61	136.97	84.88	37.38	157.58	0.19	−0.04	0.59
Kuwait	47.76	17.33	95.31	53.19	20.3	108.9	0.11	−0.08	0.39
Lebanon	42.19	15.76	84.46	54.98	22.21	107.02	0.3	−0.03	0.77
Libya	64.47	25.33	126.03	78.89	32.07	154.7	0.22	−0.01	0.52
Morocco	29.86	10.2	64.24	40.69	16.18	78.92	0.36	0.01	0.85
Oman	40.03	15.28	81.87	84.14	36.53	155.93	1.1	0.63	2.05
Palestine	85.88	33.84	167.34	124.89	49.7	244.55	0.45	0.25	0.68
Qatar	124.14	49.58	243.03	144.64	56.82	278.51	0.17	−0.01	0.38
Saudi Arabia	48.22	18.99	99.46	81.93	30.62	160.64	0.7	0.43	1.06
Sudan	53.03	21.25	102.91	66.27	26.01	129.53	0.25	0.09	0.43
Syrian Arab Republic	54.98	22.07	106.14	58.31	22.36	112.9	0.06	−0.06	0.19
Tunisia	65.4	25.76	131.93	69.69	27.93	135.41	0.07	−0.09	0.32
Türkiye	79.55	35.62	147.91	93.06	44.56	166.14	0.17	−0.04	0.51
United Arab Emirates	45.47	16.36	97.7	52.39	18.9	111.27	0.15	−0.21	0.71
Yemen	62.42	24.04	120.55	91.67	40.11	170.76	0.47	0.27	0.8
All-ages counts estimates									
North Africa and Middle East	215,015.13	89,809.31	400,651.97	511,201.35	224,528.92	926,816.17	1.38	1.15	1.8
Afghanistan	3803.54	1406.45	7628.11	19,490.27	7631.48	38,671.73	4.12	3.27	5.15
Algeria	15,209.27	5857.76	29,903.71	32,200.12	13,245.35	60,628.52	1.12	0.74	1.69
Bahrain	586.87	233.16	1152.43	1575.47	599.33	3112.95	1.68	1.37	2.09
Egypt	44,683.03	19,146.33	80,441.39	119,708.31	56,282.61	198,826.46	1.68	1.24	2.46
Iran (Islamic Republic of)	40,165.27	16,367.03	74,962.39	76,947.95	31,968.20	140,809.21	0.92	0.67	1.23
Iraq	6518.16	2477.05	13,313.40	19,804.32	8264.56	39,012.29	2.04	1.59	2.81
Jordan	2657.07	1031.48	5116.95	10,460.85	4607.20	19,421.55	2.94	2.17	4.23
Kuwait	820.84	297.82	1637.96	2473.28	944.09	5063.76	2.01	1.48	2.77
Lebanon	1262.43	471.45	2527.17	3046.28	1230.42	5929.34	1.41	0.8	2.28
Libya	2717.36	1067.69	5312.38	5420.16	2203.09	10,628.88	0.99	0.62	1.48
Morocco	7572.16	2587.55	16,288.30	15,126.74	6016.98	29,338.99	1	0.49	1.71
Oman	794.32	303.23	1624.65	3957.78	1718.09	7334.56	3.98	2.87	6.23
Palestine	1757.81	692.71	3425.33	6413.85	2552.18	12,559.02	2.65	2.13	3.21
Qatar	552.23	220.55	1081.08	4305.95	1691.69	8291.27	6.8	5.65	8.21
Saudi Arabia	7645.47	3011.28	15,771.55	30,889.71	11,546.50	60,566.96	3.04	2.41	3.91
Sudan	10,617.03	4253.56	20,604.77	28,773.99	11,293.85	56,240.17	1.71	1.37	2.09
Syrian Arab Republic	6991.78	2806.76	13,497.84	8181.32	3136.59	15,839.72	0.17	0.04	0.31
Tunisia	5460.15	2151.06	11,015.13	8253.23	3307.76	16,036.48	0.51	0.28	0.87
Türkiye	45,720.87	20,472.21	85,008.83	77,806.99	37,253.60	138,912.44	0.7	0.39	1.19
United Arab Emirates	850.64	306.04	1827.82	5046.18	1819.89	10,716.18	4.93	3.09	7.82
Yemen	8511.19	3277.48	16,436.64	30,841.81	13,495.53	57,449.30	2.62	2.14	3.44

#### 8.1.6. Anxiety disorders attributed to bullying victimization

**Figure 8.**
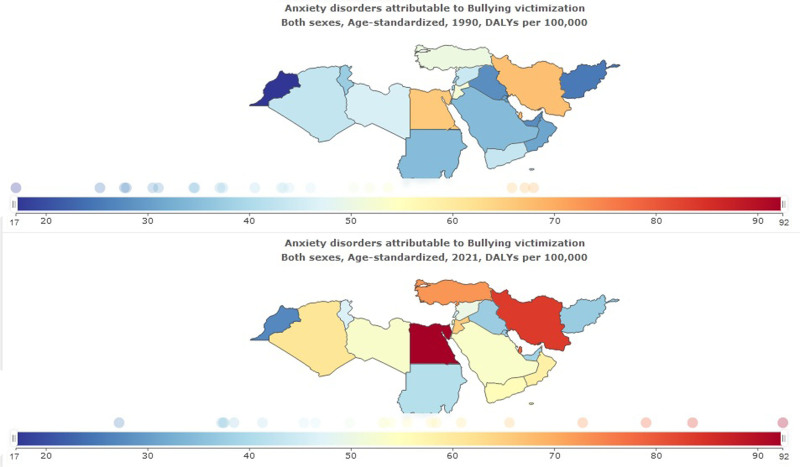
Anxiety disorders attributed to bulling victimization in North Africa and the Middle East stratified by country, 1990–2021.

DALYs of anxiety disorders attributed to bullying victimization in NAME was 410,820 (95% UI: 144,207–838,317) in 2021; compared to 1990, it showed an increase of more than 2 times 191,184 (95% UI: 67,831–401,179). Age-standardized DALYs anxiety disorders rate attributed to bullying victimization per 100,000 was 62 (95% UI: 22–126) in 2021. The highest DALYs of anxiety disorders attributed to bullying victimization in NAME was in Egypt, 92 (95% UI: 37–168) per 100,000. The lowest DALYs of depressive disorders attributed to bullying victimization in NAME was in Morocco, 27 (95% UI: 9–61) per 100,000 (Table 5, Fig. 8).

**Table 5:** DALYs of anxiety disorders attributed to bulling victimization in North Africa and the Middle East stratified by country, 1990–2021.

Location	Year								
	1990			2021			Percentage change 1990–2021		
	Value	Lower	Upper	Value	Lower	Upper	Value	Lower	Upper
Age-standardized rate estimates (per 100,000)									
North Africa and Middle East	47.56	16.01	98.5	62.47	22.23	126.81	0.31	0.17	0.57
Afghanistan	25.29	7.8	57.77	37.25	11.44	85.57	0.47	0.26	0.77
Algeria	43.1	13.26	98.37	60.82	20.22	132.4	0.41	0.19	0.81
Bahrain	53.65	16.96	118.57	57.88	18.58	122.87	0.08	−0.01	0.2
Egypt	65.81	22.74	133.42	92.46	37.03	168.01	0.4	0.14	0.87
Iran (Islamic Republic of)	67.08	23.32	133.48	83.58	29.84	159.43	0.25	0.16	0.38
Iraq	27.89	8.43	61.46	37.51	12.46	83.47	0.35	0.14	0.67
Jordan	51.82	16.08	113.54	65.57	23.17	129.95	0.27	0.03	0.69
Kuwait	30.48	9.15	66.91	37.41	12.99	78.39	0.23	0.01	0.64
Lebanon	37.08	11.52	80.11	45.33	16.08	95.93	0.22	−0.08	0.67
Libya	46.01	14.42	100.19	53.11	17.79	115.85	0.15	−0.01	0.4
Morocco	17.02	4.85	40.06	27.17	8.98	61.65	0.6	0.17	1.16
Oman	31.04	9.42	71.14	58.39	20.6	120.32	0.88	0.45	1.63
Palestine	40.52	13.06	87.29	53.96	17.45	120.5	0.33	0.15	0.53
Qatar	67.94	22.63	140.62	79.01	29.75	161.88	0.16	−0.01	0.43
Saudi Arabia	34.5	10.55	75.32	53.28	17.19	115.78	0.54	0.38	0.79
Sudan	34.62	10.41	74.26	41.27	13.16	90.22	0.19	0.04	0.4
Syrian Arab Republic	43.89	14.2	95.31	49.85	15.79	109.31	0.14	0	0.26
Tunisia	37.36	11	82.54	46.48	15.17	101.41	0.24	0.06	0.52
Türkiye	50.26	17.67	102.84	72.77	28.33	144.93	0.45	0.1	1.01
United Arab Emirates	27.69	8.47	62.65	38.47	13.99	79.82	0.39	0.06	0.88
Yemen	43.34	14.18	94.33	55.51	18.58	115.66	0.28	0.1	0.55
All-ages rate estimates (per 100,000)									
North Africa and Middle East	56.36	20	118.28	65.94	23.15	134.56	0.17	0.03	0.38
Afghanistan	29.32	9.59	65.24	46.09	14.89	101.59	0.57	0.33	0.9
Algeria	53.08	16.96	118.66	58.86	19.04	128.4	0.11	−0.1	0.41
Bahrain	63.26	18.9	142.59	59.93	17.6	131.96	−0.05	−0.14	0.05
Egypt	76.57	27.75	150.84	101.26	41.11	183.08	0.32	0.08	0.74
Iran (Islamic Republic of)	78.15	28.26	152.67	79.33	26.94	157.09	0.02	−0.12	0.15
Iraq	33.78	10.86	74.28	44.19	14.88	97.44	0.31	0.09	0.61
Jordan	67	22.26	143.33	77.56	27.79	153.11	0.16	−0.05	0.53
Kuwait	36.04	10.53	81.21	35.22	10.39	79.48	−0.02	−0.22	0.26
Lebanon	40.8	12.95	88.14	44.38	14.91	94.57	0.09	−0.2	0.51
Libya	57.34	19.45	120.68	56.56	18.68	126.24	−0.01	−0.2	0.19
Morocco	20.45	6.09	47.32	27.54	9.06	62.87	0.35	−0.01	0.82
Oman	34.97	10.77	78.98	60.7	19.49	130.01	0.74	0.31	1.44
Palestine	49.04	16.39	105.53	65.84	21.8	144.18	0.34	0.15	0.54
Qatar	76.6	23.28	167.83	81.27	23.32	173.5	0.06	−0.13	0.28
Saudi Arabia	41.58	13.02	90.97	55.96	16.51	127.55	0.35	0.13	0.57
Sudan	40.69	12.93	86.35	51.39	17.07	110.94	0.26	0.1	0.48
Syrian Arab Republic	53.8	18.33	113.04	56.33	18.91	122.92	0.05	−0.07	0.16
Tunisia	44.76	13.84	98.16	41.92	13.27	91.77	−0.06	−0.24	0.15
Türkiye	60.5	21.89	122.38	69.29	26.56	138.4	0.15	−0.12	0.6
United Arab Emirates	30.29	8.17	69.6	28.84	7.7	68.73	−0.05	−0.37	0.36
Yemen	48.2	17.46	101.31	67.78	23.52	139.98	0.41	0.21	0.69
All-ages counts estimates									
North Africa and Middle East	191,184.81	67,831.52	401,179.39	410,820.97	144,207.83	838,317.85	1.15	0.9	1.53
Afghanistan	2915.73	953.75	6487.30	14,390.71	4649.78	31,720.00	3.94	3.18	4.98
Algeria	13,421.78	4288.00	30,007.47	26,013.86	8417.26	56,750.88	0.94	0.58	1.46
Bahrain	320.39	95.70	722.18	916.62	269.26	2018.44	1.86	1.6	2.18
Egypt	42,370.07	15,357.40	83,466.56	106,956.21	43,426.58	193,378.23	1.52	1.06	2.33
Iran (Islamic Republic of)	44,621.52	16,134.39	87,174.52	67,711.63	22,996.53	134,085.32	0.52	0.31	0.71
Iraq	6221.82	2000.02	13,679.85	18,217.08	6133.75	40,169.28	1.93	1.44	2.6
Jordan	2502.90	831.66	5354.55	9558.67	3425.04	18,869.91	2.82	2.12	4.04
Kuwait	619.34	181.02	1395.67	1637.63	483.01	3696.11	1.64	1.12	2.4
Lebanon	1220.68	387.37	2637.18	2458.96	826.16	5239.49	1.01	0.48	1.79
Libya	2417.09	819.62	5086.88	3885.71	1283.35	8673.50	0.61	0.3	0.94
Morocco	5184.91	1545.16	11,999.82	10,238.55	3367.08	23,371.87	0.97	0.45	1.67
Oman	694.04	213.8	1567.19	2855.14	916.82	6115.13	3.11	2.11	4.78
Palestine	1003.83	335.57	2160.14	3381.48	1119.36	7404.53	2.37	1.89	2.85
Qatar	340.75	103.58	746.56	2419.36	694.32	5165.22	6.1	4.85	7.56
Saudi Arabia	6593.27	2064.13	14,424.08	21,100.82	6225.04	48,092.26	2.2	1.68	2.73
Sudan	8146.39	2588.29	17,288.92	22,313.04	7412.67	48,170.72	1.74	1.39	2.22
Syrian Arab Republic	6841.48	2331.56	14,375.99	7903.31	2653.43	17,246.37	0.16	0.03	0.29
Tunisia	3737.37	1155.45	8195.76	4964.79	1571.88	10,868.41	0.33	0.08	0.63
Türkiye	34,768.67	12,578.10	70,334.55	57,934.53	22,209.14	115,721.39	0.67	0.28	1.33
United Arab Emirates	566.76	152.87	1302.14	2777.42	741.35	6619.29	3.9	2.23	6.02
Yemen	6571.43	2380.94	13,812.94	22,802.28	7911.52	47,092.94	2.47	1.98	3.18

#### 8.1.7. Depressive disorders attributed to bullying victimization stratified by sex

**Figure 9.**
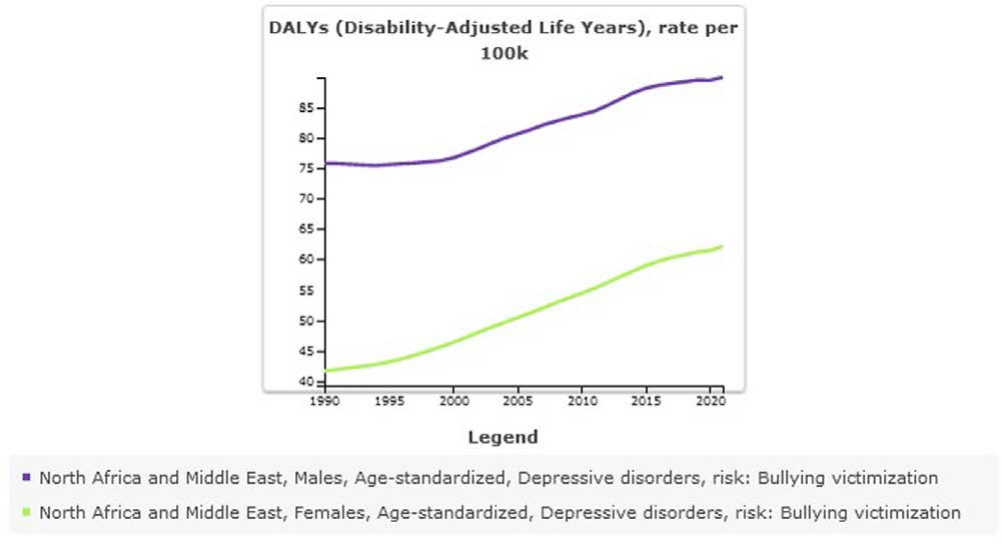
Trend of Depression disorders attributed to bulling victimization in North Africa and the Middle East stratified by country, 1990–2021.

In Male all-ages counts estimates of DALYs for depressive disorders attributed to bullying victimization were 313,274 (95% UI: 136,286–541,866] and higher than females 197,926 (95% UI: 82,579–380,717). Age-standardized DALYs for depressive disorders attributed to bullying victimization rate per 100,000 was 89 (95% UI: 39–155) in males vs 62 (95% UI: 26–119) in females (Table 6, Fig. 9). The prevalence of depressive disorders and DALYs is higher in females than in males, but the depressive disorders DALYs attributed to bullying victimization have a higher burden in males.

**Table 6:** DALYs of depressive and anxiety disorders attributed to bulling victimization in North Africa and the Middle East stratified by sex, 1990–2021.

Sex	Depressive and anxiety disorders	Year								
		1990			2021			Percentage change 1990–2021		
		Value	Lower	Upper	Value	Lower	Upper	Value	Lower	Upper
Age-standardized rate estimates (per 100,000)										
Males	Depressive disorders	75.79	31.94	136.2	89.91	39.33	155.65	0.19	0.08	0.36
Males	Anxiety disorders	62.62	22.15	123.55	74.16	28.94	139.6	0.18	0.06	0.41
Females	Depressive disorders	41.68	15.34	84.65	62.14	26.07	119.27	0.49	0.29	0.85
Females	Anxiety disorders	31.71	8.21	74.51	49.75	13.93	108.75	0.57	0.34	1.02
All-ages rate estimates (per 100,000)										
Males	Anxiety disorders	73.36	27.31	142.61	78.53	29.99	148.54	0.07	−0.05	0.25
Males	Depressive disorders	79.26	34.66	141.16	96.88	42.15	167.57	0.22	0.11	0.42
Females	Depressive disorders	46.73	17.79	94.03	66.06	27.56	127.06	0.41	0.23	0.75
Females	Anxiety disorders	38.52	10.53	89.18	52.35	14.56	114.65	0.36	0.17	0.71
All-ages counts estimates										
Males	Depressive disorders	137,702.71	60,222.39	245,247.77	313,274.46	136,286.72	541,866.38	1.28	1.06	1.64
Males	Anxiety disorders	127,455.27	47,446.35	247,757.32	253,949.77	96,993.31	480,330.13	0.99	0.76	1.32
Females	Depressive disorders	77,312.41	29,432.32	155,582.12	197,926.90	82,579.24	380,717.14	1.56	1.22	2.17
Females	Anxiety disorders	63,729.54	17,414.70	147,547.96	156,871.20	43,629.91	343,539.13	1.46	1.11	2.11

#### 8.1.8. Anxiety disorders attributed to bullying victimization stratified by sex

**Figure 10.**
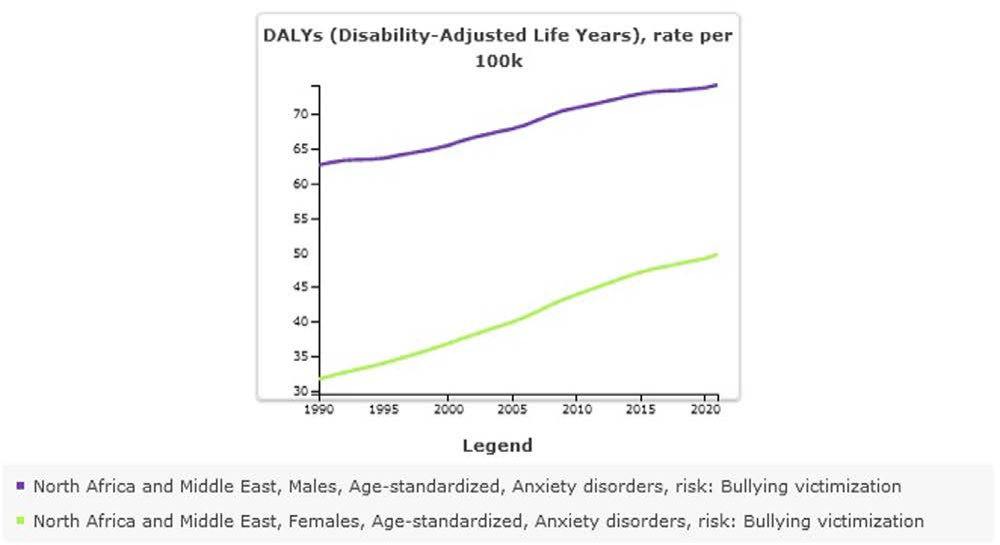
Trend of Anxiety disorders attributed to bulling victimization in North Africa and the Middle East stratified by country, 1990–2021.

In Male all-ages counts estimates of DALYs for anxiety disorders attributed to bullying victimization were 253,949 (95% UI: 96,993–480,330) and higher than females 156,871 (95% UI: 43,629–343,539). Age-standardized DALYs for anxiety disorders attributed to bullying victimization rate per 100,000 was 74 (95% UI: 28–139) in males vs 49 (95% UI: 14–108) in females (Table 6, Fig. 10). The prevalence of anxiety disorders and DALYs is higher in females than in males, but the anxiety disorders DALYs attributed to bullying victimization have a higher burden in males.

## 9. Discussion

This study investigated the burden of depressive and anxiety disorders in NAME, including 21 countries, and also investigated DALYs of depressive and anxiety disorders attributed to bullying victimization. Sex differences in the prevalence, incidence, and DALYs caused by depressive disorders were investigated and it was also shown how the trend of depressive and anxiety disorders between 1990 and 2021 was.

Depressive disorder is a serious health problem and is known to be associated with adverse health outcomes and reduced life expectancy.^[37]^ A finding obtained from this study showed that the burden of depressive disorders increased from 1990 to 2021, but in 2019, at the same time as the COVID-19 pandemic, there was a rapid growth in the prevalence of depressive disorders. This trend has been observed similarly for anxiety disorders, however, the prevalence and burden caused by anxiety disorders compared to depressive disorder disorders was higher. This finding is in line with a study that investigated the effects of COVID-19 on depressive disorder and anxiety disorders in 2020 and showed that there was an increase in depressive disorder and anxiety disorders during COVID-19.^[38]^ The period from 2019 to 2021 witnessed a significant surge in cases of depression and anxiety disorders. When compared to global statistics, the findings indicate that the Middle East and North Africa region experienced higher levels of these disorders compared to other parts of the world. An increase in depressive disorder and anxiety disorders in 2020 is associated with an increase in the infectious disease of COVID-19 and decreasing human mobility.^[38]^ Although the number of people with depressive disorder and anxiety disorders has increased sharply in 2021 compared to 1990, the standardized prevalence rate in NAME has almost a constant trend and a slight increase. Part of this increase in the number of people with depressive disorder and anxiety disorders is due to population growth and the increase in the fertility rate during the past decades,^[39]^ which has led to an increase in people with mental disorders. A part of the reasons for the increase in depressive disorder and anxiety disorders is due to geographical conditions, Gross National Income, conflicts, poor health infrastructure, war, and migration.^[39,40]^ Numerous countries within the Middle East and North Africa region are grappling with ongoing challenges such as armed conflict, widespread migration, and large-scale displacement. These pressing issues invariably contribute to heightened risks associated with mental health, as the individuals affected are often exposed to prolonged periods of instability, trauma, and uncertainty. Studies indicate a rising prevalence of mental health disorders in this region.^[41–43]^ However, changes in health structures and attention to these have been noted in recent years, and this can lead to improvements in public health, especially in the coming decades.^[44]^ At the national scale, Palestine reported the highest prevalence of depressive disorders in 2021, ranking first among 21 countries within the region. This troubling statistic underscores the significant mental health challenges faced by the Palestinian population, challenges that are deeply intertwined with broader issues such as inadequate infrastructure, ongoing conflict, the devastating impact of war, widespread displacement, and limited access to essential healthcare services.

During the past years, bullying victimization has been recognized as one of the health problem behaviors in children and adolescents.^[18]^ Findings from this study on the DALYs of depressive and anxiety disorders attributed to bullying victimization showed an increase in 2021 compared to 1990; it is consistent with the study conducted based on GBD 2019, which also showed an increase in the burden of DALYs of depressive and anxiety disorders attributed to bullying victimization.^[26]^ Being subjected to bullying has been found to correlate strongly with a heightened risk of developing suicidal thoughts. Similarly, experiences of cyberbullying have been linked to an increased likelihood of encountering various mental health challenges. These insights underscore the critical need for implementing effective programs that target and mitigate bullying behaviors in all forms. Such initiatives carry particular significance, given that mental health issues emerging during early stages of life can potentially contribute to an elevated risk of more severe psychiatric disorders later in adulthood. Addressing these concerns early on can play a pivotal role in promoting healthier emotional and psychological development over the long term.^[17]^ Although the study of sex differences in the prevalence and DALYs of depressive and anxiety disorders showed that both depressive and anxiety disorders are more common in females than males; the DALYs of depressive and anxiety disorders attributed to bullying victimization are higher in males than in females. In explaining this finding, it should be noted that although the prevalence and DALYs of depressive disorder and anxiety disorders are higher in females than in males, the exposure to bullying victimization is higher in males than in females. Previous studies also show that men are more exposed to bullying than women.^[45–47]^ Research has consistently highlighted that boys and girls are inclined to encounter distinct forms of bullying victimization, shedding light on notable gender-based disparities. Boys are often found to be at greater risk of experiencing physical bullying, involving direct confrontations such as hitting or pushing, whereas girls are more typically subjected to verbal and relational bullying.^[18]^ These forms may encompass harsh words, exclusion from social groups, or targeted rumors aimed at undermining relationships and emotional well-being. This divergence in experiences has sparked an increasing interest within the scholarly community to explore how gender influences bullying dynamics and the far-reaching impact of these behaviors on the mental, emotional, and physical health outcomes of those affected. The nature of bullying plays a significant role in shaping health outcomes for both women and men. Understanding the specific type of bullying is essential when assessing gender differences in the rates of depression and anxiety disorders linked to such experiences.^[48]^

### 9.1. Limitations

Estimates of the GBD have limitations, including the inconsistency of data availability. There are also problems with the quality and collection of primary data. The studied risk factors also have limitations, including the omission of various potentially consequential risk factors and covariates. The effects of Covid-19 are also a challenge. More details on the GBD limits are provided elsewhere.^[19,30]^ The study uses a narrow definition (school-based peer bullying only). This leaves out family, teacher, or partner abuse, which are also important. This may underestimate the real burden.

## 10. Conclusion

This study showed that depressive disorder and anxiety disorders followed a uniform trend from 1990 to 2019 in NAME, but as a result of COVID-19, there may have been a rapid growth in the prevalence of depressive disorder and anxiety disorders in 2020. There has been an increase in bullying victimization, and this has led to an increase in depressive and anxiety disorders attributed to bullying victimization, therefore, at the national level, it is necessary to make health policies on screening for mental disorders, educating children and adolescents in dealing with bullying victimization, and mental health services should be more focused.

### 10.1. Practical interventions

Practical examples of targeted interventions, such as the implementation of the Olweus Bullying Prevention Program,^[49,50]^ have the potential to significantly influence and enhance health-related policies. Programs like these are designed to systematically address concerns at multiple levels – individual, social, and institutional – by creating frameworks that prevent harmful behaviors, promote emotional well-being, and foster safe environments. For instance, the Olweus Bullying Prevention Program employs strategies that focus not only on reducing bullying incidents through clear protocols but also on improving communication between students, teachers, and parents while fostering a culture of inclusivity and mutual respect within schools. When such interventions are integrated into broader health policies, they contribute to reducing mental health issues stemming from bullying, improving overall school climate, and encouraging proactive behavior in conflict resolution. By involving various stakeholders and highlighting preventive measures, these programs can serve as models that demonstrate how localized actions have the potential to make broader systemic changes in public health approaches.

## Author contributions

**Conceptualization:** Sohrab Amiri.

**Validation:** Sohrab Amiri.

**Visualization:** Sohrab Amiri.

**Writing – original draft:** Sohrab Amiri, Seyyed Mohammad Hossein Kazemi.

**Writing – review & editing:** Sohrab Amiri, Seyyed Mohammad Hossein Kazemi.

## Supplementary Material


