# Supplementary material for: Depressive and anxiety disorders linked to bullying victimization in North Africa and the Middle East: An analysis based on sex and location
Source: Medicine (Baltimore). 2025 Nov 14;104(46):e45745. doi: 10.1097/MD.0000000000045745 (PMC12622653; doi:10.1097/MD.0000000000045745)
Supplement: Supplementary file 1 [file medi-104-e45745-s001.docx]

Supplementary Table 1. Prevalence, Incidence, DALYs, of depressive and anxiety disorders in North Africa and the Middle East stratified by sex, 1990 to 2021

| Measure | Cause | Sex | Year | | | | | | | | |
| --- | --- | --- | --- | --- | --- | --- | --- | --- | --- | --- | --- |
|  |  |  | 1990 | | | 2021 | | | Percentage change 1990-2021 | | |
|  |  |  | Value | Lower | Upper | Value | Lower | Upper | Value | Lower | Upper |
| Age-standardized Rate estimates (Per 100,000) | | | | | | | | | | | |
| Prevalence | Depressive disorders | Males | 3,467.12 | 3,040.99 | 4,033.12 | 3,949.57 | 3,445.98 | 4,569.67 | 0.14 | 0.09 | 0.19 |
|  | Depressive disorders | Females | 5,520.11 | 4,810.86 | 6,450.53 | 6,190.39 | 5,312.89 | 7,265.07 | 0.12 | 0.06 | 0.18 |
|  | Anxiety disorders | Males | 3,720.35 | 3,107.02 | 4,369.25 | 4,452.54 | 3,641.55 | 5,440.51 | 0.2 | 0.11 | 0.28 |
|  | Anxiety disorders | Females | 6,228.31 | 5,240.02 | 7,420.57 | 7,566.04 | 6,174.88 | 9,273.69 | 0.21 | 0.13 | 0.3 |
| Incidence | Depressive disorders | Males | 3,886.21 | 3,292.45 | 4,646.25 | 4,608.86 | 3,838.00 | 5,529.70 | 0.19 | 0.12 | 0.26 |
|  | Depressive disorders | Females | 6,452.98 | 5,495.62 | 7,698.83 | 7,472.52 | 6,176.78 | 9,036.59 | 0.16 | 0.09 | 0.23 |
|  | Anxiety disorders | Males | 644.26 | 535.14 | 769.69 | 778 | 637.36 | 960.09 | 0.21 | 0.12 | 0.29 |
|  | Anxiety disorders | Females | 816.16 | 674.19 | 1,023.43 | 996.95 | 799.36 | 1,286.09 | 0.22 | 0.13 | 0.31 |
| DALYs | Depressive disorders | Males | 610.4 | 414.44 | 831.7 | 709.72 | 478.73 | 974.21 | 0.16 | 0.1 | 0.23 |
|  | Depressive disorders | Females | 974.64 | 665.07 | 1,337.75 | 1,107.68 | 728.86 | 1,520.94 | 0.14 | 0.07 | 0.2 |
|  | Anxiety disorders | Males | 448.32 | 308.15 | 619.22 | 537.11 | 358.5 | 754.75 | 0.2 | 0.11 | 0.29 |
|  | Anxiety disorders | Females | 733.8 | 500.32 | 997.39 | 890.44 | 594.36 | 1,245.14 | 0.21 | 0.12 | 0.3 |
| All-ages Rate estimates (Per 100,000) | | | | | | | | | | | |
| Prevalence | Depressive disorders | Males | 2,830.99 | 2,464.02 | 3,348.52 | 3,971.75 | 3,440.36 | 4,644.29 | 0.4 | 0.33 | 0.48 |
|  | Depressive disorders | Females | 4,550.57 | 3,930.08 | 5,370.06 | 6,185.69 | 5,272.24 | 7,309.98 | 0.36 | 0.28 | 0.44 |
|  | Anxiety disorders | Males | 3,571.60 | 2,969.74 | 4,325.37 | 4,556.72 | 3,688.05 | 5,589.00 | 0.28 | 0.17 | 0.4 |
|  | Anxiety disorders | Females | 5,848.18 | 4,865.47 | 7,064.95 | 7,697.71 | 6,233.75 | 9,478.80 | 0.32 | 0.22 | 0.42 |
| Incidence | Depressive disorders | Males | 3,254.90 | 2,738.50 | 3,904.08 | 4,650.98 | 3,832.24 | 5,636.74 | 0.43 | 0.34 | 0.53 |
|  | Depressive disorders | Females | 5,450.77 | 4,558.07 | 6,649.59 | 7,494.94 | 6,142.80 | 9,158.69 | 0.38 | 0.28 | 0.48 |
|  | Anxiety disorders | Males | 668.01 | 548.06 | 811.65 | 805.13 | 651.2 | 1,000.68 | 0.21 | 0.1 | 0.32 |
|  | Anxiety disorders | Females | 859.8 | 693.99 | 1,089.32 | 1,034.38 | 824.24 | 1,341.62 | 0.2 | 0.1 | 0.3 |
| DALYs | Depressive disorders | Males | 506.61 | 342.74 | 698.73 | 718.85 | 477.82 | 979.06 | 0.42 | 0.33 | 0.51 |
|  | Depressive disorders | Females | 816.45 | 552.76 | 1,135.15 | 1,112.71 | 731.33 | 1,539.69 | 0.36 | 0.28 | 0.46 |
|  | Anxiety disorders | Males | 434.62 | 295.91 | 618.87 | 551.74 | 362.32 | 777.34 | 0.27 | 0.16 | 0.38 |
|  | Anxiety disorders | Females | 697.37 | 474.24 | 965.75 | 909.09 | 603.14 | 1,280.99 | 0.3 | 0.2 | 0.41 |
| All-ages counts estimates | | | | | | | | | | | |
| Prevalence | Depressive disorders | Males | 4,918,428.95 | 4,280,866.07 | 5,817,560.97 | 12,843,429.59 | 11,125,087.06 | 15,018,228.72 | 1.61 | 1.48 | 1.76 |
|  | Depressive disorders | Females | 7,529,219.58 | 6,502,580.16 | 8,885,115.88 | 18,534,208.56 | 15,797,224.73 | 21,902,914.93 | 1.46 | 1.32 | 1.61 |
|  | Anxiety disorders | Males | 6,205,130.27 | 5,159,488.56 | 7,514,688.01 | 14,735,050.61 | 11,926,051.13 | 18,073,129.14 | 1.37 | 1.18 | 1.6 |
|  | Anxiety disorders | Females | 9,676,199.20 | 8,050,232.38 | 11,689,423.78 | 23,064,680.80 | 18,678,188.19 | 28,401,344.39 | 1.38 | 1.2 | 1.56 |
| Incidence | Depressive disorders | Males | 5,654,908.64 | 4,757,740.13 | 6,782,772.78 | 15,039,874.31 | 12,392,293.02 | 18,227,519.29 | 1.66 | 1.49 | 1.85 |
|  | Depressive disorders | Females | 9,018,660.83 | 7,541,630.55 | 11,002,183.13 | 22,457,118.66 | 18,405,679.84 | 27,442,205.32 | 1.49 | 1.31 | 1.68 |
|  | Anxiety disorders | Males | 1,160,565.57 | 952,181.50 | 1,410,119.26 | 2,603,541.44 | 2,105,771.15 | 3,235,897.72 | 1.24 | 1.05 | 1.46 |
|  | Anxiety disorders | Females | 1,422,601.40 | 1,148,248.37 | 1,802,356.18 | 3,099,305.97 | 2,469,682.17 | 4,019,908.93 | 1.18 | 1 | 1.36 |
| DALYs | Depressive disorders | Males | 880,159.73 | 595,453.81 | 1,213,933.90 | 2,324,531.79 | 1,545,116.52 | 3,165,968.61 | 1.64 | 1.48 | 1.82 |
|  | Depressive disorders | Females | 1,350,869.83 | 914,585.85 | 1,878,183.68 | 3,334,020.70 | 2,191,297.13 | 4,613,389.32 | 1.47 | 1.31 | 1.64 |
|  | Anxiety disorders | Males | 755,088.39 | 514,097.38 | 1,075,188.68 | 1,784,165.88 | 1,171,630.95 | 2,513,690.38 | 1.36 | 1.17 | 1.58 |
|  | Anxiety disorders | Females | 1,153,836.53 | 784,655.10 | 1,597,904.34 | 2,723,901.55 | 1,807,191.68 | 3,838,236.08 | 1.36 | 1.18 | 1.55 |
